# Effect of Multidisciplinary Case Conferences on Physician Decision Making: Breast Diagnostic Rounds

**DOI:** 10.7759/cureus.895

**Published:** 2016-11-24

**Authors:** Tianne J Foster, Antoine Bouchard-Fortier, Ivo A Olivotto, May Lynn Quan

**Affiliations:** 1 Surgical Oncology, University of Calgary/Tom Baker Cancer Center; 2 Department of Oncology, University of Calgary/Tom Baker Cancer Center

**Keywords:** multidisciplinary, case conference, tumour board, breast cancer, decision-making

## Abstract

Purpose: To evaluate the utility of multidisciplinary case conferences (MCCs) on physician decision making in benign and malignant breast disease management.

Methods:  Patients with interesting or challenging diagnostic or management issues were discussed at biweekly diagnostic breast MCCs. Prior to discussion, a clinical summary and intended management plan prior to the MCC was presented. For each case, diagnostic images/histopathology were centrally reviewed after which group discussion achieved a management consensus which was documented prospectively. Initial management plans were compared to the post-MCC consensus. A change in a management plan was defined as a consensus plan different from the pre-MCC plan or no definite plan prior to the MCC.

Results:  From November 2014 to December 2015, 76 patients (43 malignant and 33 benign diagnoses) were discussed in 19 MCCs. All cases presented resulted in a consensus management recommendation. Thirty-one case discussions (41%) resulted in a changed management plan (20 malignant and 11 benign diagnoses). Management changes included avoidance of immediate surgery (9% of cases), change in the type of surgery (5%), non-invasive investigation to invasive/surgical intervention (7%), and detection of a new suspicious lesion (1%).

Conclusion: MCCs had a substantial impact on physician decision making. Management plans changed in 41% of cases presented, the majority due to new/clarified diagnostic information. Presentation of cases at MCCs should be encouraged, especially for challenging diagnostic or management issues regarding malignant or benign breast diagnoses.

## Introduction

Breast disease management is complex and multidisciplinary. An effective diagnosis is based on clinical-radiology-pathology correlation which depends on regular open communication between disciplines [1]. Cancer Care Ontario (CCO) proposed standards for multidisciplinary case conferences (MCCs) and described a framework including recognition of primary and secondary functions. The primary function of an MCC is to ensure all diagnostic tests and treatment options are considered for individual patients [2]. Secondary functions include continuing education to aid in appropriate and timely referrals, contribute to research, and ensure quality care [2].

In Calgary, Alberta, breast diagnostic MCCs were instituted bi-weekly in 2010 to review cases with benign or malignant breast pathology with challenging diagnostic or management issues. Many studies have been conducted on the impact of MCCs for patients with malignant breast pathology. However, to our knowledge, no studies have assessed the value of MCCs for patients with both benign and malignant pathology. This is a report of a prospective evaluation of the utility of MCCs on physician decision making for patients with benign and malignant breast diseases.

## Materials and methods

A prospective evaluation was conducted of MCCs that occurred every second Wednesday afternoon for one hour, from November 5th, 2014 to December 9th, 2015 but excluding July to September 2015. The MCCs were video-linked between six sites in the city of Calgary, Alberta, Canada. Patients with diagnostic or management issues were selected for presentation by the attending physician, generally their surgeon. The cases included benign or malignant pathology and may have been new diagnoses or recurrent disease. MCC attendees included surgeons (general surgeons and surgical oncologists), subspecialty trained radiologists and pathologists, medical and radiation oncologists, general practitioners with an oncology focus, nurse coordinators, and trainees. A summary of the patient’s clinical history was presented and the question(s) for the MCC were described by the presenting physician who also stated his/her intended management if the MCC had not been available. Diagnostic images and histopathology were reviewed by a breast expert radiologist and pathologist, respectively, so that all cases had a 'central' review. The pre-MCC intended management was recorded and compared to the MCC consensus management recommendation. A management “change” was defined as a difference compared to the pre-MCC plan or if there was no definite management plan prior to the MCC.

## Results

Nineteen biweekly MCCs occurred during which 76 patients were discussed. One to eight patients (median = 4) were discussed at each MCC. A minimum of one radiologist, pathologist, and surgeon were present at each MCC. Radiation and medical oncologist attendance was variable, but there were complementary weekly city-wide oncology management rounds at which patients' oncologic management may have been discussed. The range of participants at any given round was 5-15. Trainee and non-physician attendance was not recorded. Attendance was recorded mainly for the purposes of continuing medical education credit, and thus attendance from video-linked sites was not well captured.

Of the 76 cases, 33 had only benign pathology, and 43 had malignant diagnoses. Five benign cases had a prior history of breast cancer. After review of the diagnostic information and MCC discussion, management changes were recommended for 31 (41%) patients (Table 1). Sixty-two of the cases (82%) had a proposed plan prior to the MCC; whereas for 14 (18%), the management decision was dependent on the discussion. Of the 31 cases with a management plan change, 20 had malignant diagnoses (65%), and 11 were benign (35%). Fourteen cases (45%) changed due to new or clarified information from diagnostic imaging, 9 cases (29%) changed due to new or clarified details from histopathology, and 8 cases (26%) changed after both diagnostic imaging and histopathology review and group discussion.

**Table 1:** Changes in Management as a Result of a Review of Diagnostic Imaging Films/Histopathology and Discussion Abbreviations: SLNB - sentinel lymph node biopsy, CT - computed tomography, BCS - breast conserving surgery

Pre-MCC plan	Post-MCC consensus plan	Malignant or benign disease	No. of cases
BCS	Mastectomy	Malignant (3)	3
BCS	Biopsy	Malignant	1
BCS	MRI, neoadjuvant chemotherapy + Herceptin	Malignant	1
Biopsy right lesion	Biopsy right lesion and suspicious left lesion	Malignant	1
Follow /no excision	Surgical excision	Benign	1
Follow	Re-biopsy	Benign	1
Image localization	Major duct excision, no localization	Benign	1
No SLNB	Perform SLNB	Malignant	1
Plan dependent on MCC consensus	Biopsy	Malignant (1) Benign (1)	2
Plan dependent on MCC consensus	BCS	Malignant	1
Plan dependent on MCC consensus	Mastectomy	Malignant	1
Plan dependent on MCC consensus	Surgical excision	Malignant (2)	2
Plan dependent on MCC consensus	Re-excision of margins	Malignant	1
Plan dependent on MCC consensus	No biopsy/follow	Benign (2)	2
Plan dependent on MCC consensus	Further imaging	Benign (3)	3
Plan dependent on MCC consensus	Radiotherapy	Malignant (2)	2
Radiotherapy	Biopsy/external histopathology slide review	Benign	1
Refer for surgery	Repeat biopsy	Malignant	1
Refer for surgery	Radiotherapy	Malignant	1
Segmental mastectomy and SLNB	Re-imaging with CT	Malignant	1
SLNB and ?needle core or ?excisional biopsy	Excisional biopsy, no SLNB	Malignant	1
Surgical excision	No surgical excision	Malignant	1
Surgical excision	Vacuum assisted biopsy	Benign	1

The changes that took place involved avoidance of immediate surgery in 7/31 (23%) cases, change in the type of surgery 4/31 (13%), non-invasive investigation to invasive/surgical intervention 5/31 (16%), and detection of a new suspicious lesion in one case (3%) (Table 2). Our results demonstrated a 9% avoidance of surgery after MCC discussion. In a lower percentage of cases (7%), the post-MCC plan determined that surgical/invasive intervention was necessary as opposed to the pre-MCC intent for non-invasive investigation (Table 2). In those five cases, a review of both diagnostic imaging and histopathology determined that a sentinel lymph node biopsy (SLNB) should be provided, and a second patient should be re-biopsied. Review of histology with discussion resulted in a recommendation for surgical excision; a biopsy was recommended instead of a previously planned radiotherapy, and in one case, imaging review with discussion resulted in a plan for surgical excision. In 5% of the cases, the type of surgery changed; three cases changed from breast conserving surgery to mastectomy, and one case recommended a sentinel lymph node biopsy (SLNB) not be performed. In one case, a new suspicious lesion was revealed for which a biopsy was recommended (Table 2).

**Table 2:** Overall Changes in Management Plans after Multidisciplinary Conference Case Reviews

Post-MCC consensus plan	No. of cases	Percent of total cases (%)	Based on review/discussion of
No immediate surgery	7	9.2	Imaging alone (5) Histopathology and Imaging (2)
Change in type of surgery	4	5.2	Imaging alone (3) Histopathology and Imaging (1)
Surgical/invasive intervention recommended	5	6.6	Imaging alone (1) Histopathology alone (2) Histopathology and Imaging (2)
Biopsy new suspicious lesion	1	1.3	Imaging alone

## Discussion

Of the 76 cases evaluated as part of this prospective study, a change in management recommendation occurred in 41% following multidisciplinary discussion. By presenting selected, challenging cases rather than all consecutive new diagnoses, there may have been a greater likelihood for management plan changes. However, even when individual patient management plans did not change, others have reported a benefit from MCCs regarding validation and building consensus about management of future patients [3-5].

In the United States, breast centers have been established since 1979 conveying an importance for multidisciplinary care in breast disease. Many breast centers conform to the standards set forth by the National Accreditation Program for Breast Centers [6-7]. Similarly in Europe, the European Accreditation of Breast Units section within the European Society of Mastology provides guidelines for breast centers in Europe [6]. On both sides of the Atlantic, conducting regular, interdisciplinary cancer case conferences is one of the standards required for a breast center to maintain accreditation [6, 8]. Uniform Canadian national standards may be a valuable resource.

In Canada, the use of MCCs and their structure is variable between centers. In 2006, Cancer Care Ontario made recommendations regarding the structure, function, and purpose of MCCs. In 2008, minimum criteria were established for Ontario sites conducting MCCs [2, 9].  Any site treating more than 35 cancer patients within a specific disease site was required to hold an MCC in the specific subspecialty [2]. Cancer Care Ontario also established guidelines for the meeting format, team members and attendees, their respective roles and responsibilities, and institutional requirements such as video conferencing and teleconferencing capabilities [2]. Other jurisdictions do not have published specific standards for MCCs.

MCCs have been shown to impact diagnosis, patient care planning, and compliance with clinical practice guidelines [10-14]. A systematic review of 27 studies published from 1995 to 2015 demonstrated management plan changes in 19% to 34.5% of cases discussed in oncology MCCs [3, 10]. Studies with a retrospective design reported a higher incidence of changes (52%) [3, 15]. In the current study, a change rate of 41% is comparable to other prospective studies [3].

During the MCC review, physicians discussed concordance of findings between histopathology and diagnostic imaging while determining their treatment plan. Much of the discussion involved clarification of the current imaging or histopathology and may not have resulted in a specific change to the report itself. In a recent study, Prakash et al., [1] described the outcomes of weekly, multidisciplinary breast radiology-histopathology correlation conferences for percutaneous breast core needle biopsies which resulted in a change in management to avoid surgery in 2.1% of cases (29/1387) and detected additional cancers at a rate of 2.2 per 1000 cases [1]. Improved concordance after core biopsy review for benign cases has also been demonstrated [16]. Our review demonstrated that presentation at the MCC helped avoid surgery in 9% of the cases and found a new lesion in one patient (1.3%).

Many studies have found that MCCs positively impact patient care, and a few have shown survival benefits associated with MCC presentation [17-22]. A study from the United Kingdom retrospectively compared patient outcomes and breast cancer survival over five years in one health board without MCCs to centers in a health board which introduced an MCC review process. It was found that breast cancer mortality was 18% lower in the health board implementing MCCs as compared to the health board without MCCs [17]. It was also found that within the health board implementing MCCs, there was less variation in breast cancer survival rates between hospitals [17].

Regardless of these benefits, some still question the efficiency and impact of MCCs [5, 23]. In 2015, the American Society of Clinical Oncology (ASCO) conducted an international survey of a cohort of ASCO members located outside of the United States regarding multidisciplinary cancer conferences at their centers [5]. Four hundred nine physicians responded with their reasons for attending MCCs, which included obtaining treatment recommendations (89% of respondents) or participating in discussions (86%). However, in these scenarios it was common for all patients with a new diagnosis of breast cancer to be presented at the meeting (49% of respondents); whereas 34.5% of respondents attended MCCs where only selected new breast cancer cases were presented, and 16.5% of respondents stated that there were no selection criteria for the discussed breast cancer cases [5]. Overall, the case discussions at multidisciplinary meetings were geared toward cancer diagnoses. To date, there have not been any studies aside from correlation meetings, to our knowledge, which have evaluated the performance and utility of MCCs that included benign and malignant breast disease management. Presenting benign breast disease cases was useful in the present study as over a third of such cases had a management change.

Attendance, another common issue for MCCs, is variable although many physicians report that they believe MCCs benefit patients [5, 24-25]. In the current study, the majority of cases were presented by surgeons 77% (24/76), with minimal attendance from medical oncology who presented one case. Other cases were presented by radiation oncology, pathology, and a general practitioner with an oncology focus. Increased participation and case presentation by all breast cancer specialists should be encouraged.

In order to improve attendance and MCC function, many have desired for an effective moderator and improved time management [2-3, 5, 23, 25]. When time is scarce among busy physicians, MCCs may be attended sporadically by those who do not find the meetings well organized and effectively time managed. Although not formally assessed, presenting select cases non-consecutively as opposed to a consecutive presentation of all breast cancer cases was perceived to allow more effective management of meeting times [25]. A limitation of our study was that the impact of the changes to the management plans on patient outcomes was not able to be determined.

MCC discussion not only benefited patients but also incited discussion of policies and care processes. This improved interdisciplinary communication. For example, initially the practice of pathologists was to uniformly recommend excision of certain benign lesions (i.e., atypical ductal hyperplasia or papilloma) found on core biopsy within the body of the pathology report. This practice was restrictive, and given the multiple discussions during MCCs on whether or not to excise such lesions, it was agreed by the group that changing the wording on the pathology reports to “recommendation for surgical consultation” was preferred. This improved interdisciplinary communication among participants in MCCs has been noted as a benefit of multidisciplinary meetings [26-27].

## Conclusions

This study demonstrated that multidisciplinary case conferences had a substantial impact on physician decision making for both benign and malignant breast disease. Nearly half of the case discussions (31 of 76) resulted in a change in the clinical recommendation. The majority of management changes were based on new/clarified diagnostic imaging or histopathology information. Presentation of cases at MCCs should be encouraged, especially for challenging diagnostic or management issues.
